# Supplementary material for: Mechanisms governing the pioneering and redistribution capabilities of the non-classical pioneer PU.1
Source: Nat Commun. 2020 Jan 21;11:402. doi: 10.1038/s41467-019-13960-2 (PMC6972792; doi:10.1038/s41467-019-13960-2)
Supplement: Supplementary file 7 — Source data [file 41467_2019_13960_MOESM7_ESM.zip › Source_Data/Figure6/Figure6B_MotifScanOutput/FLI1_mutPU.1/homerResults/motif48.similar.html]

motif48

## Information for motif48

C
G
A
T
C
T
A
G
T
A
G
C
C
T
A
G
T
A
G
C
T
G
C
A
C
A
G
T
A
T
C
G
A
G
T
C
A
C
T
G
A
G
T
C
  
Reverse Opposite:  

T
C
A
G
T
G
A
C
T
C
A
G
T
A
G
C
G
T
C
A
A
C
G
T
A
T
C
G
G
A
T
C
A
T
C
G
G
A
T
C
G
C
T
A
  

|  |  |
| --- | --- |
| p-value: | 1e-20 |
| log p-value: | -4.725e+01 |
| Information Content per bp: | 1.680 |
| Number of Target Sequences with motif | 262.0 |
| Percentage of Target Sequences with motif | 2.55% |
| Number of Background Sequences with motif | 457.9 |
| Percentage of Background Sequences with motif | 1.20% |
| Average Position of motif in Targets | 100.1 +/- 59.4bp |
| Average Position of motif in Background | 90.5 +/- 62.8bp |
| Strand Bias (log2 ratio + to - strand density) | -0.1 |
| Multiplicity (# of sites on avg that occur together) | 1.09 |
| Motif File: | file (matrix) reverse opposite |

### Similar de novo motifs found

|  |  |  |  |  |  |  |  |
| --- | --- | --- | --- | --- | --- | --- | --- |
| Rank | Match Score | Redundant Motif | P-value | log P-value | % of Targets | % of Background | Motif file |
| 1 | 0.981 | A T G C A G C T C T A G T A G C C T A G A G T C T G C A A C G T A C T G A G T C A C T G G A T C | 1e-20 | -46.408185 | 2.47% | 1.15% | motif file (matrix) |
| 2 | 0.973 | C G A T A T G C A G C T T C A G T G A C T C A G T A G C T G C A A C G T T C A G G A T C A T C G A G T C T C G A | 1e-19 | -43.983864 | 2.32% | 1.08% | motif file (matrix) |
| 3 | 0.972 | A G C T C T A G T G A C T C A G A G T C T G C A A C G T A C T G A G T C A C T G G A T C T C G A T A C G | 1e-18 | -43.453884 | 2.35% | 1.11% | motif file (matrix) |
| 4 | 0.873 | C T A G T G A C T C A G T A G C G C T A G C A T T A C G G A T C C A T G | 1e-16 | -38.322502 | 3.28% | 1.85% | motif file (matrix) |
| 5 | 0.950 | G C A T C T A G T G A C T C A G G T A C G T C A C A G T A C T G G A T C A C T G | 1e-16 | -37.208050 | 3.40% | 1.96% | motif file (matrix) |
